# Impact of the Luding Earthquake on the Area of Potentially Suitable Habitat for *Batrachuperus* Species in the Gongga Mountain National Nature Reserve

**DOI:** 10.3390/ani15020235

**Published:** 2025-01-16

**Authors:** Xinlong Song, Xiuying Liu, Xiaoao Zheng, Jian Song, Zhangqiang You, Jianli Xiong

**Affiliations:** 1 Ecological Security and Protection Key Laboratory of Sichuan Province, Mianyang Normal University, Mianyang 621000, China; syz202203@163.com (X.S.); songjian7654321@163.com (J.S.); youzq@mtc.edu.cn (Z.Y.); 2 School of Resources and Environmental Engineering, Mianyang Normal University, Mianyang 621000, China; csfulxy@126.com; 3 Sichuan Gongga Mountains National Nature Reserve, Kangding 626000, China; z1449872551@163.com

**Keywords:** distribution, endangered species, environmental variables, maxent, salamander

## Abstract

Members of the genus *Batrachuperus* are endemic to China and include seven described species, five of which are nationally protected salamanders in China. The Gongga Mountain National Nature Reserve (GGMNNR) is rich in animal and plant resources, including the *Batrachuperus* species. On 5 September 2022, the GGMNNR experienced the Luding earthquake, and this provided an opportunity to quantify the impact of a catastrophic events on the area of potentially suitable habitat for a rare species in the GGMNNR. The main objective of this study was to evaluate the impact of the Luding earthquake on the area of potentially suitable habitat for the *Batrachuperus* species by combining Maxent modeling, remote sensing image processing, and GIS methods. The results showed that precipitation, temperature, and slope were the most important variables affecting the distribution of the *Batrachuperus* species. Currently, suitable habitats accounted for 40.67% (1664.10 km^2^) of the studied area (4091.10 km^2^), and these areas were mainly distributed in the northern, western, and southern portions of the GGMNNR. The destruction caused by the earthquake accounted for 4.87% (199.34 km^2^) of the studied area, and only 1.97% (32.78 km^2^) of the suitable habitat of the *Batrachuperus* species was destroyed. These results indicated that although the Luding earthquake affected the habitat of the *Batrachuperus* species in the GGMNNR, its effect was small. These findings enhance our understanding of the distribution of the *Batrachuperus* species and the effect of the Luding earthquake on biodiversity in the GGMNNR.

## Simple Summary

Understanding the impact of earthquakes on habitat suitability is crucial for formulating effective protection and management measures for endangered species with limited dispersal capacities, as well as the conservation of their habitats. The Gongga Mountain National Nature Reserve (GGMNNR) is rich in animal and plant resources. On 5 September 2022, the GGMNNR experienced the Luding earthquake, and this provided an opportunity to quantify the impact of a catastrophic event on the area of potentially suitable habitat for a rare species. Here, we evaluated the effect of the Luding earthquake on the area of potentially suitable habitat for the *Batrachuperus* species in the GGMNNR based on predictions of the current potentially suitable habitat area. These findings enhance our understanding of the distribution of *Batrachuperus* species and the effect of the Luding earthquake on the biodiversity in the GGMNNR.

## 1. Introduction

Habitat, the environment in which a particular species can accomplish its life cycle [1], plays a crucial role in species survival and reproduction [2]. Suitable habitats provide species with adequate living environments, which include sufficient food, shelter, and protection. Habitat loss, fragmentation, isolation, degradation, and destruction may lead to declines in local populations, reductions in genetic diversity, and even the extinction of a species [3,4,5,6,7].

The habitat of a species can be affected by various factors, such as human activity, natural disasters, and climate change. Natural disasters include wildfires, floods, storms, volcanic eruptions, earthquakes, and tsunamis. Earthquakes are one of the most destructive natural disasters. They are sudden seismic movements of great intensity caused by the abrupt liberation of energy due to the deformation of the lithosphere, which propagates as seismic waves in all directions [8]. An earthquake is considered destructive when it exceeds 6.0 degrees on the Richter scale [9], which can lead to changes in the original landscape and the destruction/loss of terrestrial vegetation through their large destructive force and subsequent geohazards, such as mountain floods, debris flow, and landslides [10,11,12,13,14]. This can ultimately damage an ecosystem’s structure and function, habitat quality, and local populations. For example, the Chi-Chi earthquake (M = 7.3) in central Taiwan disrupted the hydrology of streams and ponds, which caused dramatic decreases in *Rana swinhoana* populations [15]. The Wenchuan earthquake (M = 8.0) in Sichuan Province, China, severely damaged 15.2% of the habitat of three amphibians (*Batrachuperus tibetanus*, *B. pinchonii*, and *Amolops mantzorum*) in the earthquake-affected area [16]. Understanding the impact of earthquakes on habitat suitability for an endangered species with limited dispersal capacities is crucial for formulating effective protection and management measures for preserving these species and conserving their habitats.

Mountain salamanders (genus *Batrachuperus*) are endemic to China, and seven species are currently described, including *B. pinchonii*, *B. tibetanus*, *B. karlschmidti*, *B. yenyuanensis*, *B. londongensis*, *B. taibaiensis*, and *B. daochengensis* [17]. These are cold-water species that reside in montane streams throughout the year [18], and they are primarily distributed in the mountainous areas of western China, including the Sichuan, Shanxi, Gansu, Xizang, and Yunnan provinces [19,20,21]. Three (*B. pinchonii*, *B. tibetanus*, and *B. karlschmidti*) of them are currently classified as vulnerable; two (*B. yenyuanensis* and *B. londongensis*) of them are classified as endangered by the IUCN due to over-exploitation, timber and plant harvesting, infrastructure development, pollution, and mining/energy production [22]; and five (*B. pinchonii*, *B. tibetanus*, *B. karlschmidti*, *B. yenyuanensis*, and *B. londongensis*) of them are listed as class II nationally key protected species in China.

**Figure 1:**
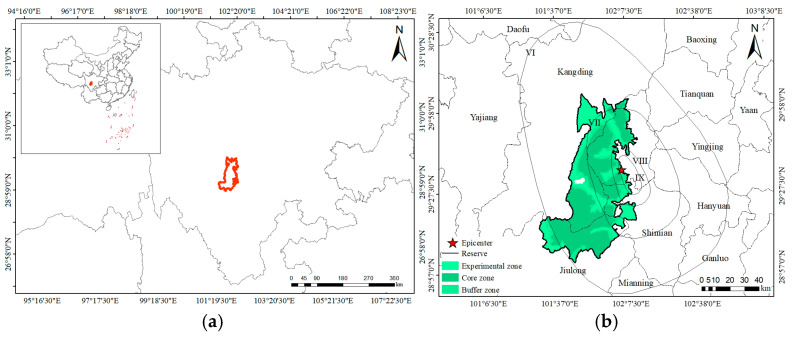
Geographic location of the Gongga Mountain National Nature Reserve (**a**) and the intensity distribution of the Luding earthquake (**b**).

The Gongga Mountain National Nature Reserve (GGMNNR), one of the world’s global biodiversity hotspots [23], is located in the center of the Daxue Mountains on the southeastern edge of the Qinghai-Tibetan Plateau (Figure 1a), and it is a priority area for biodiversity conservation in China. The *Batrachuperus* species is abundant in the GGMNNR, but the taxonomy of this species remains unresolved in this reserve, as mitochondrial DNA (mtDNA) data have revealed that hybridization has occurred in some areas. The species involved include *B. tibetanus*, *B. karlschmidti*, *B. yenyuanensis*, and *B. daochengensis* (Xiong et al., unpublished data). On 5 September 2022, an earthquake with a magnitude of 6.8 and a maximum intensity of IX degrees struck Luding County, Garze Tibetan Autonomous Prefecture, Sichuan Province in southwestern China (hereinafter referred to as the Luding earthquake). The epicenter (29.59° N, 102.08° E at a depth of 16 km) was located approximately 7 km southwest of Moxi Town (Figure 1b). This earthquake resulted in a great loss of human life and property, including 93 deaths and 25 missing people [24], and tens of thousands of buildings were damaged [25,26]. Several studies on this earthquake have been conducted, including comprehensive analyses [27], as well as analyses of the rupture process [28]; surface deformation, fault rupture, and coseismic geohazards [29]; and earthquake-induced landslides [30,31]. However, no studies to date have evaluated the impact of this earthquake on the area of suitable habitat for a rare species. The epicenter was located in the experimental zones of the GGMNNR, 20 km from Gongga Mountain. Most areas of the GGMNNR were located in zone VI (Figure 1b). This major disturbance has provided an opportunity to evaluate the impact of a catastrophic event on the area of potentially suitable habitat for a rare species in the GGMNNR.

In this study, the impacts of the Luding earthquake on the potentially suitable habitat for the *Batrachuperus* species in the GGMNNR were explored. The environmental features of the optimal habitats for closely related species were expected to be similar given that the rate of niche evolution was not fast enough to eliminate all signs of common ancestry [32,33]. The environmental similarities of the habitats of the *Batrachuperus* species have been demonstrated by our field surveys. Thus, the taxonomy of this species in the GGMNNR has not been examined, and this species has been referred to as the *Batrachuperus* species. The aims of this study were to (1) define the current area of potentially suitable habitat for the *Batrachuperus* species in the GGMNNR using the Maxent model; (2) identify the impact range of the Luding earthquake in the GGMNNR; and (3) evaluate the impact of the Luding earthquake on the current area of potentially suitable habitat for the *Batrachuperus* species. Based on the magnitude and damage of the Luding earthquake, we predicted that the Luding earthquake had a large impact on the potential suitable habitat of the *Batrachuperus* species.

## 2. Materials and Methods

### 2.1. Study Area

The GGMNNR (29°01′~30°05′ N, 101°29′~102°12′ E) is situated in the center of the Daxue Mountains of the Hengduan Mountains on the southeastern edge of the Qinghai—Tibetan Plateau, and the altitude of the GGMNNR ranges from 1200 m to 7556 m (the summit of Gongga Mountain). This reserve was established in 1996 to protect the forest ecosystems of the Daxue Mountains (mainly, the Gongga Mountains), various rare and wild animal and plant resources, and low-altitude modern glaciers [34]. The total area of the GGMNNR is approximately 4091.10 km^2^, and it features a temperate plateau climate with annual precipitation that varies from 800 to 900 mm. The dry season runs from early November to May, and the rainy season runs from June to October. There are 3795 species of plants (including 6 class I and 11 class II nationally key protected species in China) and 587 species of animals (including 16 class I and 62 class II nationally key protected species in China).

### 2.2. Prediction of the Current Area of Potentially Suitable Habitat

#### 2.2.1. Data Collection

The species presence data were obtained from our original field survey, published literature, and the Global Biodiversity Information Facility website (GBIF, http://www.gbif.org, accessed on 10 August 2024). During the filed survey, a presence point was recorded when an individual (adult or larva) of the *Batrachuperus* species was found. Individuals from the same population were recorded only once. The published literatures containing presence points include studies by Fu et al. [19], Liu et al. [35], and Tang et al. [36]. A total of 74 occurrence records were collected, including 60 from the field survey, 12 from the published literature, and 2 from the GBIF.

Based on the biological characteristics of the *Batrachuperus* species, 30 environmental variables (Table 1) were initially selected, which included bioclimatic, geographical, vegetational, anthropogenic, and meteorological factors. The bioclimatic variable data (Bio1–Bio19) were downloaded from the WorldClim climate database (http://www.Worldclim.org/, accessed on 10 August 2024), with a spatial resolution of 30 arc-seconds and approximately 1 km^2^ [37]. The geographical data included aspect, slope, altitude, and distance to water (dis_water). The DEM layers at a resolution of 30 m were sourced from the Geospatial Data Cloud platform of the Computer Network Information Center, Chinese Academy of Sciences (https://www.gscloud.cn, accessed on 10 August 2024), and the slope and aspect were calculated from the DEM layers using ArcGIS 10.8 software (ESRI Inc., Redlands, CA, USA). The water source layers were downloaded from the National Geomatics Center of China (https://www.ngcc.cn, accessed on 10 August 2024), and the Dis_water was calculated as the Euclidean distance to water derived from the water source layers in ArcGIS 10.8 software (ESRI Inc., Redlands, CA, USA). The vegetation data included the vegetation fractional cover (VFC) and vegetation type (VT), which were derived from the Global Map Data Archives (https://globalmaps.github.io/ptc.html, accessed on 10 August 2024) and Resources and Environmental Science Data Center (https://www.resdc.cn/, accessed on 10 August 2024), respectively. The anthropogenic data included the human footprint (HFP) and distance to road source (Dis_road). The HFP layer was obtained from the NASA Socio-economic Data and Applications Center (https://www.earthdata.nasa.gov/centers/sedac-daac, accessed on 10 August 2024). The road layers were obtained from the National Geomatics Center of China (https://www.ngcc.cn, accessed on 10 August 2024), and the Dis_road data were calculated as the Euclidean distance to roads derived from the road layers in ArcGIS 10.8 software (ESRI Inc., Redlands, CA, USA). The meteorological data comprised the mean annual cloud cover (Mannual), seasonal cloud cover concentration (Mseasonal), and spatial variability in cloud cover (Mspatial), which were downloaded from EarthEnv (https://www.Earthenv.org/cloud, accessed on 10 August 2024).

#### 2.2.2. Data Processing

**Figure 2:**
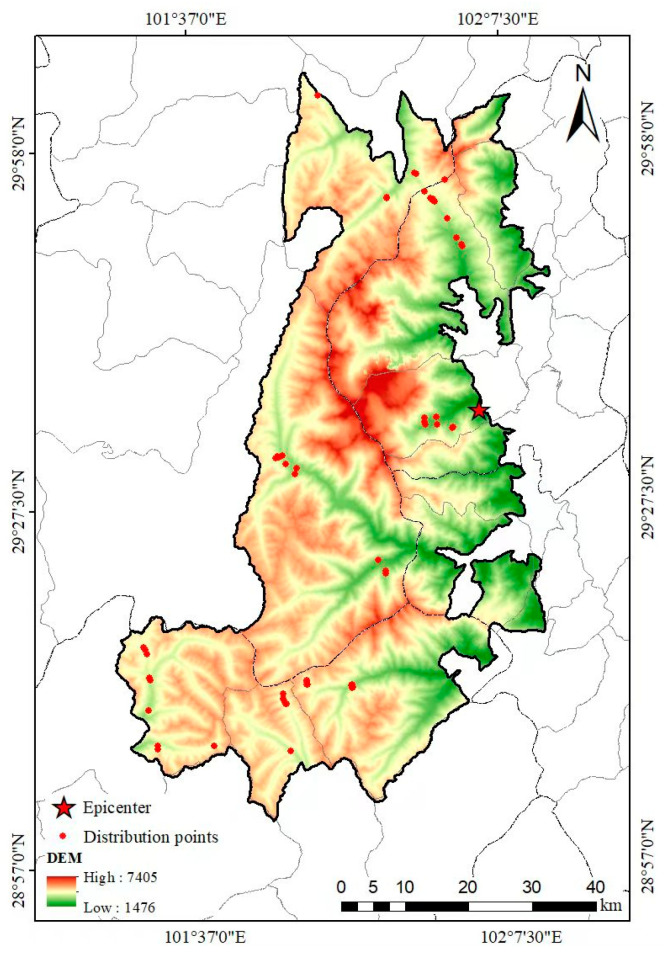
Distribution points of the *Batrachuperus* species in the Gongga Mountain National Nature Reserve after excluding autocorrelations.

To avoid the spatial clustering and pseudoreplication of the occurrence points and reduce the overfitting of the model, the occurrence data were thinned using SDMToolbox (version 2.4) [38] in ArcGIS 10.8 software (ESRI Inc., Redlands, CA, USA). Given that salamanders have low vagility rates, with movement ranges of less than 1 km [39], the thinning distance was set to 1 km. After thinning, 33 occurrence points were retained (Figure 2) and stored in CSV format.

**Figure 3:**
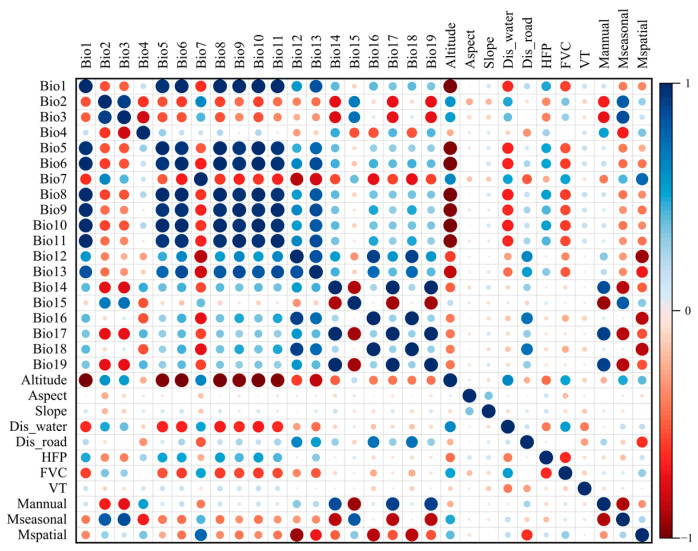
Correlation matrix of the environmental variables. The descriptions of each abbreviation were listed in Table 1. The positive correlations are displayed in blue and the negative correlations in a red color. The color intensity and the sizes of the circles are proportional to the correlation coefficients.

The coordinate system was unified (GCS_WGS_1984) and the spatial resolution was resampled (30 s, ~1 km^2^) in ArcGIS 10.8 software (ESRI Inc., Redlands, CA, USA, and the environmental variable layers were clipped according to the range of the GGMNNR. The raster data were uniformly converted into ASCII format as required by Maxent 3.4.4. To reduce multicollinearity and increase the accuracy of the prediction results, the environmental variables with high correlations but low contribution rates were removed before the model analyses [40]. A correlation analysis was performed using ENMTools.pl (https://github.com/danlwarren/ENMTools, accessed on 15 August 2024), and the correlation coefficients (Figure 3) were calculated [41]. The contribution rate of each variable was accessed in Maxent 3.4.4 by the jackknife method using the occurrence points and the 30 environmental variables [42]. The variables with very high correlations (|PCCs| ≥ 0.8) but low contribution rates (<1%) were removed [43]. If two variables were highly correlated, the variable that contributed the most to the model was retained. A total of fourteen environmental variables were finally selected to construct the final models, which included five bioclimatic, two meteorological, two vegetation, two human activity, and three geographical variables (Table 2).

#### 2.2.3. Model Parameter Optimization

**Figure 4:**
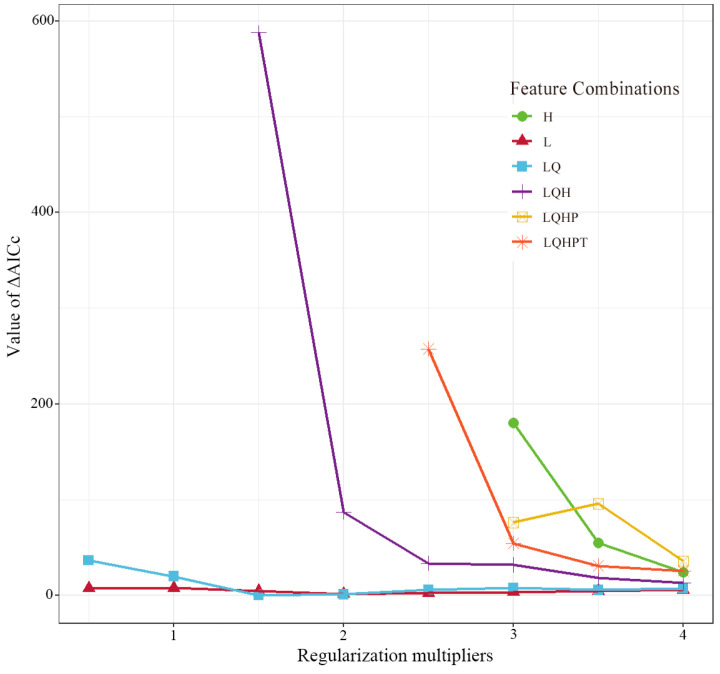
ΔAICc values of the Maxent models under different regularization multipliers (RMs) and feature combinations (FCs).

The use of default parameters can lead to over-fitting and high omission rates when implementing Maxent models [44]. The regularization multiplier (RM) and feature combination multiplier (FC; the combination of the feature types) parameters in Maxent were used to optimize the analysis of the model. There are the following five feature types in Maxent models: linear (L), quadratic (Q), hinge (H), product (P), and threshold (T). The delta from the Akaike information criterion with the correction (ΔAICc) values of the RM and FC were computed in R 4.3.3 software using the ENMeval package [45]. A smaller ΔAICc value indicates lower model complexity and a higher performance [45]. In this study, the range of RM was set from 0.5 to 4.0 at 0.5 increments, and six FC types (i.e., L, LQ, H, LQH, LQHP, and LQHPT) were selected. When the optimal model parameters were RM = 1.5 and FC = LQ, the ΔAICc value (= 0) was the lowest (Figure 4).

#### 2.2.4. Model Construction

The occurrence data, environmental variables, and the optimized model parameters were input into Maxent 3.4.4 (https://biodiversityinformatics.amnh.org, accessed on 20 August 2024). Twenty-five percent of the occurrence points were randomly selected for testing, and the rest were considered for training [46]. A total of 10,000 points were randomly selected as background points, and bootstrap was selected as the replicated run type, with 10 repetitions. The final simulation result was the mean of 10 repetitions.

#### 2.2.5. Model Accuracy Evaluation

The area under the curve (AUC) value of the receiver operating characteristic (ROC) curve was used to evaluate the model’s accuracy and effectiveness. The AUC is a threshold-independent technique used to evaluate model performance by differentiating presence from absence [47]. The AUC value, which ranges from 0 to 1, is the area enclosed by the ROC curve and abscissa, and its size represents the accuracy of the model’s prediction results [48]. A larger value indicates higher model accuracy, as well as higher credibility. Models have high prediction accuracy when their AUC values are greater than 0.8 [49]. If an AUC value is greater than 0.9, the prediction accuracy of the model is extremely high [48].

#### 2.2.6. Classification of Potentially Suitable Habitats

The outputs of the Maxent model were saved in ASCII format and then converted into raster format using ArcGIS 10.8 software (ESRI Inc., Redlands, CA, USA). In the final model, the suitability index ranged from 0 to 1, which represented the potential degree of habitat suitability for the species. The suitable habitats were reclassified and divided into the following four levels by the natural breaks (Jenks) method: habitats with high, moderate, and low suitability and an unsuitable habitat [50]. The area and proportion of a potentially suitable habitat for each level were calculated, and a distribution map of the suitable habitat was drawn.

### 2.3. Effect of the Luding Earthquake on the Current Suitable Habitat Area

#### 2.3.1. Images Collection

The TM images (Landsat 8) were downloaded from the Geospatial Data Cloud platform of the Computer Network Information Center, Chinese Academy of Sciences (https://www.gscloud.cn, accessed on 10 August 2024) at a 30 m resolution. To account for the differences in the land use types caused by phenology, the TM images from 11 November 2021 were selected to represent the pre-earthquake period and those from 20 November 2022 were selected to represent the post-earthquake period.

#### 2.3.2. Image Processing

Due to changes in the sensing position, the uneven sensing medium, atmospheric refraction, and the relief of terrain induced some deformations and errors in the TM images [51], and the TM images both before and after the earthquake were projected to GCS_WGS_1984 while atmospheric correction and radiometric calibration were carried out in ENVI 5.3. The processed TM images were clipped according to the ranges of the GGMNNR.

Land uses in the TM images both before and after the earthquake were classified into four types, including forest (trees and shrubs), grassland, bare land, and others, using the iterative self-organizing data analysis technique (ISODATA) in ArcGIS10.8 software (ESRI Inc., Redlands, CA, USA). Next, categories were defined and subcategories were fused based on the results of the unsupervised classification, which was, in turn, based on the field investigations and known types of the ground objects.

#### 2.3.3. Determination of the Regions Destroyed by the Earthquake

The damage of the earthquake to the reserve was mainly manifested by the destruction of surficial vegetation, which resulted in the transformation of forest and grassland to bare land. Thus, the bare land of the post-earthquake period included the bare land of the pre-earthquake period and the transformed area, and the region destroyed by the earthquake refers to the transformed area. A map with the area destroyed by the earthquake was obtained by calculating the area of bare land before and after the earthquake.

#### 2.3.4. Analysis of the Impact of the Luding Earthquake on the Area of Potentially Suitable Habitat

The effect of the earthquake on the area of potentially suitable habitat was analyzed based on the superposition of the suitable habitat layer and the region destroyed by the earthquake. The spatial distribution characteristics of the area of suitable habitat destroyed were analyzed. All of these steps were carried out in ArcGIS10.8 software (ESRI Inc., Redlands, CA, USA).

## 3. Results

### 3.1. Prediction Accuracy Evaluation

**Figure 5:**
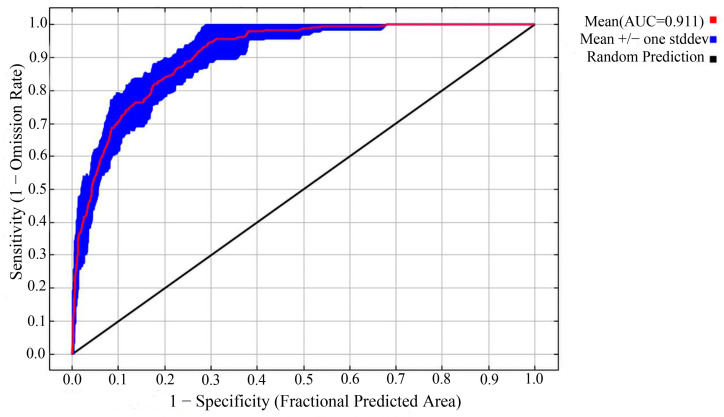
Receiver operating characteristic (ROC) curve and AUC values.

Under the optimal model parameters, an AUC value of 0.911 ± 0.019 was obtained for the ROC of the prediction results from Maxent (Figure 5), which indicated that the models had high predictive performance.

### 3.2. Importance of Environmental Variables

**Figure 6:**
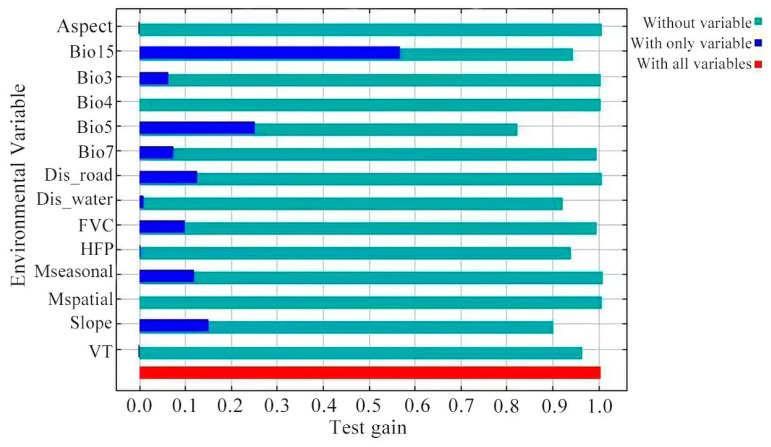
Jackknife of test gain for the environmental variables.

**Figure 7:**
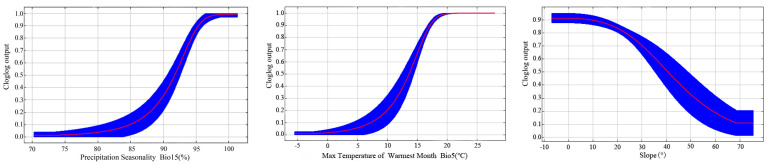
The response curves of the major climate factors (red is the response curve and blue is the standard error).

The importance of the environmental variables was evaluated using percent contribution (PC) and permutation importance (PI) (Table 2). The three environmental variables with the highest PC values were the precipitation seasonality (Bio15, 25.4%), maximum temperature of the warmest month (Bio5, 24.0%), and slope degree (slope, 12.1%), which accounted for 61.5% of the cumulative contribution. The three environmental variables with the highest PI were the maximum temperature of the warmest month (Bio5, 40.8%), precipitation seasonality (Bio15, 18.9%), and slope degree (slope, 11.1%), which accounted for 70.8% of the total. According to the jackknife test (Figure 6), the three most important environmental variables were the precipitation seasonality (Bio15), maximum temperature of the warmest month (Bio5), and slope degree (slope). The results from these methods indicated that the primary environmental variables influencing the distribution of the potentially suitable habitat for the *Batrachuperus* species in the GGMNNR were the precipitation seasonality (Bio15), maximum temperature of the warmest month (Bio5), and slope degree (slope). Based on the response curves of the major environmental variables (Figure 7), the optimal ranges for the precipitation seasonality (Bio15), maximum temperature of the warmest month (Bio5), and slope degree (slope) for the *Batrachuperus* species in the GGMNNR were > 92%, 14–22 °C, and 0–35°, respectively.

### 3.3. Current Area of Potentially Suitable Habitat

**Figure 8:**
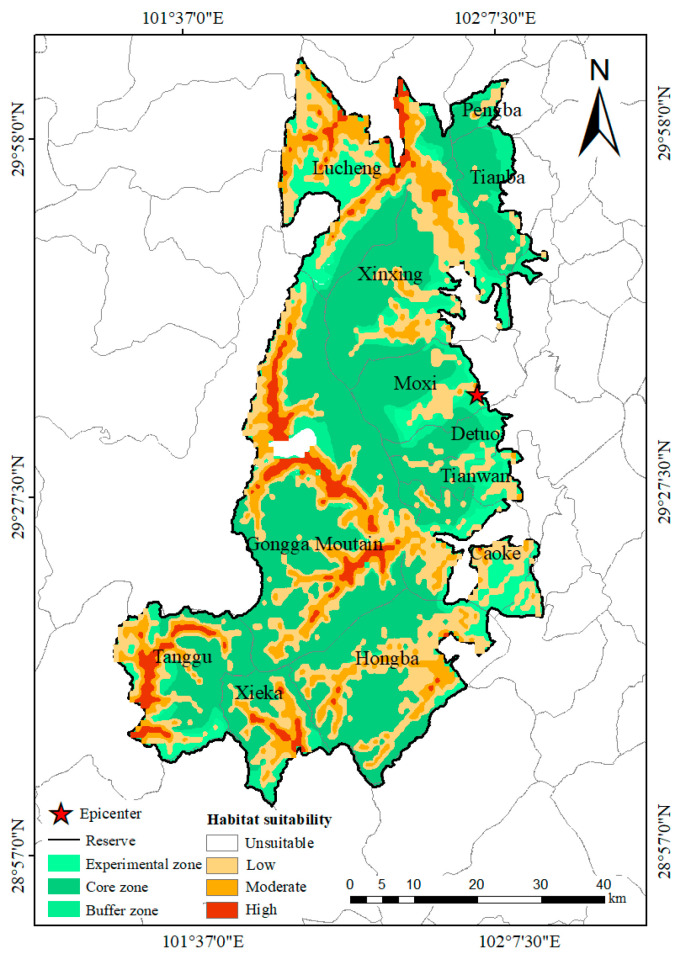
Distribution of the current potentially suitable habitats of the *Batrachuperus* species in the Gongga Mountain National Nature Reserve.

The current area of potentially suitable habitat for the *Batrachuperus* species was primarily distributed in the northern, western, and southern GGMNNR, including Lucheng Town and the Xinxing, Tanggu, Xieka, Hongba, Caoke, and Gongga Mountain townships (Figure 8). The total suitable area was approximately 1664.10 km^2^, which accounted for 40.67% of the studied area. Specifically, the highly suitable area covered approximately 179.67 km^2^, representing 4.39% of the study area, and it was predominantly distributed in Lucheng Town and the Xinxing, Gongga Mountain, Tanggu, and Xieka townships. The moderately suitable area, which had a ring shape and encompassed the highly suitable area, contained 413.78 km^2^, accounting for 10.11% of the study area. The low-suitability area surrounded both the highly and moderately suitable areas, and it was also distributed in scattered areas in the Pengba, Tianba, Tianwan townships and Moxi Town, covering 1070.65 km^2^ and representing 26.17% of the study area. The unsuitable area was 2427.34 km^2^, which represented 59.33% of the study area. The area of suitable habitat in the core, buffer, and experimental zone was 516.36 km^2^ (25.28, 108.11, and 382.97 km^2^ for habitats with high, moderate, and low suitability, respectively), 317.37 km^2^ (23.46, 68.66, and 225.25 km^2^ for habitats with high, moderate, and low suitability, respectively), and 830.37 km^2^ (130.93, 237.01, and 462.43 km^2^ for habitats with high, moderate, and low suitability, respectively), respectively (Table 3).

### 3.4. Spatial Distribution Characteristics of the Regions Destroyed by the Earthquake

**Figure 9:**
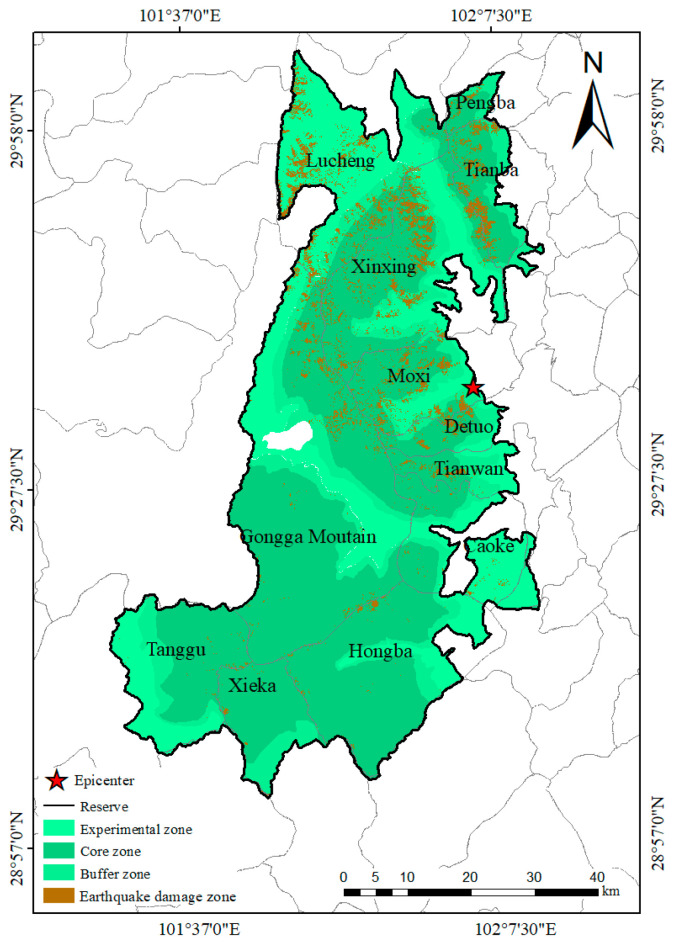
Distribution of the earthquake-destroyed regions in the Gongga Mountain National Nature Reserve.

The earthquake and its secondary disasters destroyed a total of 199.34 km^2^ of surface vegetation, accounting for 4.87% of the study area. These destroyed areas (Table 4) included forest (69.27 km^2^), grassland (98.79 km^2^), and others (31.28 km^2^), and they were mainly distributed in Lucheng Town, the Xinxing township, the Gongga Mountain township, the Pengba township, the Tianba township, Moxi Town, the Detuo township, and the Tianwan Township (Figure 9). Approximately 31.79% (63.38 km^2^), 60.51% (120.62 km^2^), 7.46% (14.88 km^2^), and 0.19% (0.38 km^2^) of the destroyed regions (Table 4) were located in the seismic intensity zones VI, VII, VIII, and IX, respectively. Based on the functional areas of the reserve, 62.75% (125.08 km^2^), 14.16% (28.23 km^2^), and 23.09% (46.03 km^2^) of the destroyed regions (Table 4) were situated in the core, buffer, and experimental zones, respectively.

### 3.5. Impact of the Earthquake on the Potentially Suitable Habitat

**Figure 10:**
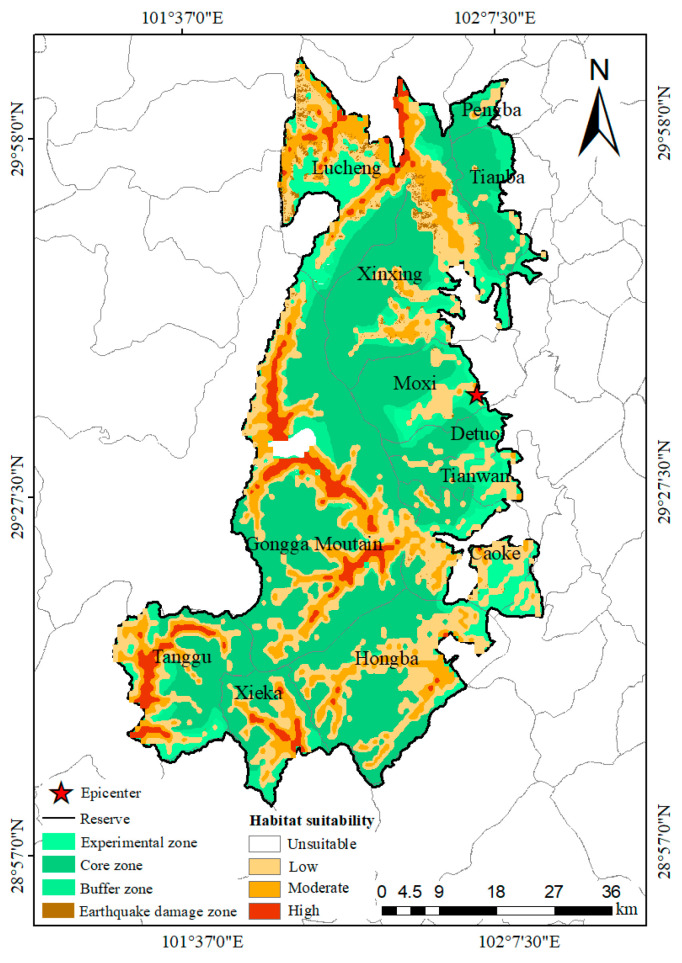
Distribution of the destroyed habitat of the *Batrachuperus* species in the Gongga Mountain National Nature Reserve.

A total of 1.97% (32.78 km^2^) of suitable habitat was destroyed by the earthquake, including 0.01 km^2^ of highly suitable habitat, 3.21 km^2^ of moderately suitable habitat, and 29.56 km^2^ of habitat with low suitability. These destroyed habitats were mainly located in Lucheng Town, the Xinxing township, the Gongga Mountain township, Moxi Town, the Caoke township, and the Tianwan township (Figure 10).

## 4. Discussion

This study explored the impact of the Luding earthquake on the area of potentially suitable habitat for the *Batrachuperus* species in the GGMNNR. The results showed that the precipitation, temperature, and slope were the most important variables limiting the distribution of the *Batrachuperus* species. The results did not support our hypothesis as the Luding earthquake had a small effect on the potentially suitable habitat of the *Batrachuperus* species.

The results from the environmental factor analyses indicated that the precipitation seasonality (Bio15), maximum temperature of the warmest month (Bio5), and slope degree (Slope) were the significant variables determining the distribution of potentially suitable habitats for the *Batrachuperus* species in the GGMNNR. The *Batrachuperus* species is water-dwelling salamander that is known to reside in montane streams throughout the year [18]. Excessive precipitation seasonality may increase stream flow, which leaves salamanders vulnerable to being washed away. In contrast, a lack of precipitation can reduce stream flow or even lead to the complete drying of streams, preventing salamanders from completing their life cycles. The slope degree is a factor affecting the velocity of a montane stream. Higher slopes indicate faster velocities, which make salamanders vulnerable to being washed away and reduce the number of plankton on which salamanders can prey. Temperature is the main environmental factor limiting ectothermic organisms, especially amphibians [52]. The *Batrachuperus* species is a cold-water species [18] and highly reliant on cold environments. High-temperature environments can increase the metabolic rates of salamanders [53]; inhibit their anti-predation behaviors, foraging, and reproductive activities [4,54,55]; and degrade mountain stream environments by reducing the amount of dissolved oxygen [56].

The prediction results showed that 40.60% (1661.10 km^2^) of the study area comprised potentially suitable habitat (including habitats with high, moderate, and low suitability) for the *Batrachuperus* species, which indicated that habitat resources are relatively abundant in the GGMNNR. However, most of the highly (154.39 km^2^, representing 85.93% of the highly suitable habitat) and moderately (305.67 km^2^, accounting for 73.87% of the moderately suitable habitat) suitable habitats were distributed in the buffer and experimental zones (Figure 8). These areas are threatened by human activities, such as grazing and farming, and thus, they require more conservation attention.

A total of 4.87% (199.34 km^2^) of the study area was destroyed by the Luding earthquake, indicating that this earthquake had a small effect on surficial vegetation. The epicenter was located in the experimental zones of the GGMNNR, close to the boundary of the reserve. Additionally, most destroyed areas were located in the seismic intensity zones VI (31.79%) and VII (60.51%) and not in zones VIII, and IX. This may have contributed to the low amount of damage caused by the earthquake.

In the areas destroyed by the earthquake, only 1.97% (32.78 km^2^) comprised suitable habitats for the *Batrachuperus* species, and most (29.56 km^2^, representing 90.18% of the areas destroyed by the earthquake) comprised habitats of low suitability (Figure 10). Compared with the Wenchuan earthquake, which destroyed 15.2% of the habitats of three amphibians in the earthquake-affected area [16], the results of this study indicated that the Luding earthquake had a small effect on the suitable habitat of the *Batrachuperus* species. As shown in Figure 8, Figure 9 and Figure 10, most of the suitable habitats for the *Batrachuperus* species were located outside the areas destroyed by the earthquake. This might have contributed to the presence of the *Batrachuperus* species in mountain streams, but the earthquake mainly affected the middle and upper parts of the mountain. The earthquake also affected the water flow of the mountain streams and destroyed the mountain stream environments; however, this was not observed in our field survey. Second, the *Batrachuperus* species is a water-dwelling salamander [18]. Our field surveys indicated that the *Batrachuperus* species prefers to inhabit vegetated montane streams with many stones of different sizes, and they usually remain under stones. The banks of the streams are mostly mud, and they are rich in vegetation, such as trees, shrubs, and herbs. The vegetative root systems can decrease the probability of the occurrence of landslides by enhancing soil reinforcement and shear strength and preventing soil erosion by holding the soil in place and improving water absorption [57]. This may stem from the small area of suitable habitat destroyed by the earthquake.

We assessed the impact of the Luding earthquake on the current area of potentially suitable habitat for the *Batrachuperus* species in the GGMNNR. However, many rare species inhabit the GGMNNR, and the impact of the Luding earthquake on the habitat suitability for these species remains unclear. Additional studies are needed to evaluate the impact of the Luding earthquake on the current areas of potentially suitable habitat for other rare species.

## 5. Conclusions

To our knowledge, few studies have examined the effects of earthquakes on the areas of suitable habitat for salamanders. Our study sheds light on the effect of the Luding earthquake on the current area of suitable habitat for the *Batrachuperus* species in the GGMNNR based on predictions of the current suitable habitat. Suitable habitats for the *Batrachuperus* species are relatively abundant in the GGMNNR. The Luding earthquake affected the current distribution of suitable habitats for the *Batrachuperus* species, but the impact on the current areas of suitable habitat was small. However, to better protect these salamanders and their habitats, changes in the areas of suitable habitat caused by human activities, climate change, and natural disasters need to be monitored. These findings enhance our understanding of the distribution of the *Batrachuperus* species and the effect of the Luding earthquake on biodiversity, and they provide baseline information for the conservation of these salamanders in the GGMNNR.

## Figures and Tables

**Table 1:** Environment variables for the Maxent modeling.

Type	Code	Description/Unit
Climatic factor	Bio1	Annual mean temperature/°C
	Bio2	Mean diurnal range/°C
	Bio3	Isothermality (Bio2/Bio7) (×100)
	Bio4	Temperature seasonality
	Bio5	Max temperature of warmest month/°C
	Bio6	Min temperature of coldest month/°C
	Bio7	Range of annual temperature (Bio5-Bio6)/°C
	Bio8	Mean temperature of wettest quarter/°C
	Bio9	Mean temperature of dryest quarter/°C
	Bio10	Mean temperature of warmest quarter/°C
	Bio11	Mean temperature of coldest quarter/°C
	Bio12	Annual average precipitation/mm
	Bio13	Precipitation of wettest month/mm
	Bio14	Precipitation of dryest month/mm
	Bio15	Precipitation seasonality (coefficient of variable)/%
	Bio16	Precipitation in wettest quarter/mm
	Bio17	Precipitation of dryest quarter/mm
	Bio18	Precipitation of warmest quarter/mm
	Bio19	Precipitation of coldest quarter/mm
Geographical factor	Aspect	Slope aspect/°
	Slope	Slope degree/°
	Altitude	Altitude/m
	Dis_water	Distance to water/m
Vegetational factor	VFC	Vegetation fractional cover
	VT	Vegetation type
Anthropogenic factor	HFP	Human footprint
	Dis_road	Distance to road/m
Meteorological factor	Mannual	Mean annual cloud cover
	Mseasonal	Seasonal cloud cover concentration
	Mspatial	Spatial variability in cloud cover

**Table 2:** Contribution and permutation importance of the environmental variables in the Maxent models.

Code	Environmental Variable	Percentage Contribution (%)	Permutation Importance (%)
Bio15	Precipitation seasonality (coefficient of variable)	25.4	18.9
Bio5	Max temperature of warmest month	24	40.8
Slope	Slope degree	12.1	11.1
Mseasonl	Cloud-cover seasonal concentration	11.1	2.7
Dis_road	Distance to road	10.6	4.6
Bio7	Range of annual temperature (Bio5-Bio6)	4.2	6.5
Dis_water	Distance to water	3.9	4
HFP	Human footprint	3.3	3.3
VT	Vegetation type	1.9	5.3
FVC	Fractional vegetation cover	1.5	0.3
Aspect	Aspect	1.3	1.7
Mspatial	Cloud-cover spatial variability	0.7	0.5
Bio3	Isothermality (Bio2/Bio7) (×100)	0.1	0.2
Bio4	Temperature seasonality	0	0.1

**Table 3:** Potentially suitable habitat areas in the different functional zones (km^2^).

Habitat Grade	Core	Buffer	Experimental	Total
Low	382.97	225.25	462.43	1070.65
Moderate	108.11	68.66	237.01	413.78
High	25.28	23.46	130.93	179.67

**Table 4:** Earthquake-destroyed areas under different land types, intensity zones, and functional zones (km^2^).

Type	Land Type			Intensity Zone				Functional Zone		
Category	Forest	Grassland	Other	VI	VII	VIII	IX	Core	Buffer	Experimental
Area	69.27	98.79	31.28	63.38	120.62	14.88	0.38	125.08	28.23	46.03

## Data Availability

The data are contained within the article.
